# Atlas construction and spatial normalisation to facilitate radiation-induced late effects research in childhood cancer

**DOI:** 10.1088/1361-6560/abf010

**Published:** 2021-05-04

**Authors:** Catarina Veiga, Pei Lim, Virginia Marin Anaya, Edward Chandy, Reem Ahmad, Derek D’Souza, Mark Gaze, Syed Moinuddin, Jennifer Gains

**Affiliations:** 1 Centre for Medical Image Computing, Department of Medical Physics and Biomedical Engineering, University College London, London, United Kingdom; 2 Department of Oncology, University College London Hospital NHS Foundation Trust, London, United Kingdom; 3 Radiotherapy Physics Services, University College London Hospital NHS Foundation Trust, London, United Kingdom; 4 UCL Cancer Institute, University College London, London, United Kingdom; 5 Radiotherapy, University College London Hospital NHS Foundation Trust, London, United Kingdom

**Keywords:** childhood cancer, computed tomography, radiotherapy, image registration, spatial normalisation, anatomical atlas

## Abstract

Reducing radiation-induced side effects is one of the most important challenges in paediatric cancer treatment. Recently, there has been growing interest in using spatial normalisation to enable voxel-based analysis of radiation-induced toxicities in a variety of patient groups. The need to consider three-dimensional distribution of doses, rather than dose-volume histograms, is desirable but not yet explored in paediatric populations. In this paper, we investigate the feasibility of atlas construction and spatial normalisation in paediatric radiotherapy. We used planning computed tomography (CT) scans from twenty paediatric patients historically treated with craniospinal irradiation to generate a template CT that is suitable for spatial normalisation. This childhood cancer population representative template was constructed using groupwise image registration. An independent set of 53 subjects from a variety of childhood malignancies was then used to assess the quality of the propagation of new subjects to this common reference space using deformable image registration (i.e. spatial normalisation). The method was evaluated in terms of overall image similarity metrics, contour similarity and preservation of dose-volume properties. After spatial normalisation, we report a dice similarity coefficient of 0.95 ± 0.05, 0.85 ± 0.04, 0.96 ± 0.01, 0.91 ± 0.03, 0.83 ± 0.06 and 0.65 ± 0.16 for brain and spinal canal, ocular globes, lungs, liver, kidneys and bladder. We then demonstrated the potential advantages of an atlas-based approach to study the risk of second malignant neoplasms after radiotherapy. Our findings indicate satisfactory mapping between a heterogeneous group of patients and the template CT. The poorest performance was for organs in the abdominal and pelvic region, likely due to respiratory and physiological motion and to the highly deformable nature of abdominal organs. More specialised algorithms should be explored in the future to improve mapping in these regions. This study is the first step toward voxel-based analysis in radiation-induced toxicities following paediatric radiotherapy.

## 1. Introduction

Radiation therapy (RT) is currently used to treat 40%–50% of childhood cancer cases in the UK (The Royal College of Radiologists 2019). While the radiation is precisely targeted to destroy the cancer cells, it may also damage surrounding healthy cells leading to sequalae that can appear years to decades after treatment (Armstrong *et al* 2009, Arain *et al* 2015). The higher risk of radiation-induced late effects in children is linked to the increased sensitivity of developing tissues, where radiation induces both organ damage and impairment of maturational processes (Paulino *et al* 2010). Furthermore, with current survival rates reaching 75% at 10 years (Cancer Research UK 2015), most paediatric patients become long term survivors, allowing for late effects to manifest. The long term harmful effects of radiotherapy include infertility, impaired physical growth and pubertal development (Schwartz 1999), renal problems (Skinner 2018), neurocognitive deficits (Roddy and Mueller 2016), as well as a range of other life-threatening issues. Second cancers are the leading cause of mortality in long term survivors, followed by cardiac and pulmonary death (Armstrong *et al* 2009). Reducing radiation-induced side effects is one of the most important ongoing challenges in paediatric cancer treatment, but there is a lack of evidence-based dose/volume guidelines to inform treatment planning. This has recently been recognised internationally with the establishment of the Paediatric Normal Tissue Effects in the Clinic task force (Constine *et al* 2019), which seeks to increase knowledge about paediatric radiotherapy dose constraints using published data.

Predictive models of radiation-induced side effects are a powerful tool to guide treatment planning and clinical decision-making. The development and validation of treatment toxicity models is however very challenging, and when considering paediatric populations specific obstacles must be addressed (Constine *et al* 2019). Radiation dose to volume is the key predictive factor of radiation-induced effects. In adults, radiation-induced effects occur mostly in organs within the radiotherapy target volumes. In contrast, for children organs and tissues outside the target volume are also important, as side-effects may develop in different regions receiving lower doses at different timescales. It is common for treatments to encompass large volumes in comparison to children’s body size (e.g. in craniospinal irradiation (CSI)), meaning a wider range of organs and tissues can receive a significant radiation dose. Smaller bodies also cause organs to be closer to the high-dose regions, increasing dose due to secondary radiation. Tissues and organs which are not directly irradiated may still have a long-term risk of radiation-induced second cancers, as result of leakage and scattered radiation (Xu *et al* 2008, Harrison 2013). Additionally, the quality of toxicity models depends on the quantity and quality of the data. Collating large datasets for individual cancer types is challenging, as childhood cancers are both rare and heterogeneous (Pappo *et al* 2015). Moreover, routine clinical data is not detailed and has limited delineations of organs and tissues; likewise, anatomy remote from the target volume is not usually imaged. To achieve larger sample sizes, it is desirable to identify methodologies that can leverage all clinically existing anatomical and dosimetric information from this heterogeneous cohort, including partial data. This can potentially be achieved by finding solutions to group patients according to organ at risk and not disease diagnosis (Constine *et al* 2019). Radiotherapy delivery is rapidly evolving, with advanced techniques such as intensity modulated radiotherapy, intensity modulated arc-therapy (IMAT), helical tomotherapy, passive scattering proton therapy, and pencil beam scanning proton therapy (PBS-PT) becoming more accessible (Sterzing *et al* 2009, Mesbah *et al* 2011, Ludmir *et al* 2018, Padovani *et al* 2019). These not only change the characteristics of dose distribution in healthy tissues (for example, low dose bath in IMAT and biological effectiveness of protons), but also make it even more challenging to achieve larger sample sizes for assessment per modality.

To address these challenges and facilitate analysis of complex 3D imaging and treatment data from heterogeneous patient groups, a possible solution to is to define a 3D common reference space and normalise spatial information from individuals of the patient group into it. Image registration is used to propagate spatial data (such as 3D imaging information and dosimetry) from the individuals onto the common reference space, which may be defined as a representative subject or unbiased population atlases (Joshi *et al* 2004, Ghosh *et al* 2010). Spatial normalisation allows one to move from region-of-interest to voxel-based analysis, which is particularly desirable in radiotherapy research to understand dose-toxicity relationships (Palma *et al* 2020). Spatial normalisation preserves the 3D information of the dose distributions (Monti *et al* 2018), unlike traditional techniques that simplify volumetric dose into 2D dose-volume histograms (DVHs). It is an advantageous approach that allows one to identify heterogeneous regional radiosensitivity (i.e. sub-volumes of organs and tissues) while not relying on *a priori* definition of volumes (Palma *et al* 2020). The need to consider the actual spatial distribution of doses, rather than organ DVHs, is recognised in late normal tissue damage research for paediatric populations (Trott 2017). Spatial normalisation in radiotherapy has become a topic of interest in recent years, with recent studies focusing on radiation-induced side-effects on prostate, head and neck and lung (Acosta *et al* 2013, Dréan *et al* 2016, Palma *et al* 2016, Monti *et al* 2017, Beasley *et al* 2018, Mylona *et al* 2019, 2020, Marcello *et al* 2020, McWilliam *et al* 2020) and to predict outcomes (Ibragimov *et al* 2019).

In this work, we investigate the feasibility of atlas construction and spatial normalisation in paediatric radiotherapy to enable voxel-based analysis of radiation-induced toxicities. The methodology was developed to serve as a framework to facilitate the development, validation, and clinical translation of radiotherapy-induced late effects models in childhood cancer patients. The atlas-based approach allows one to spatially standardise a heterogenous population in an unbiased way, while preserving localised spatial anatomical, functional and dosimetric information.

## 2. Methods and materials

### 2.1. Paediatric atlas construction

**Figure 1.**
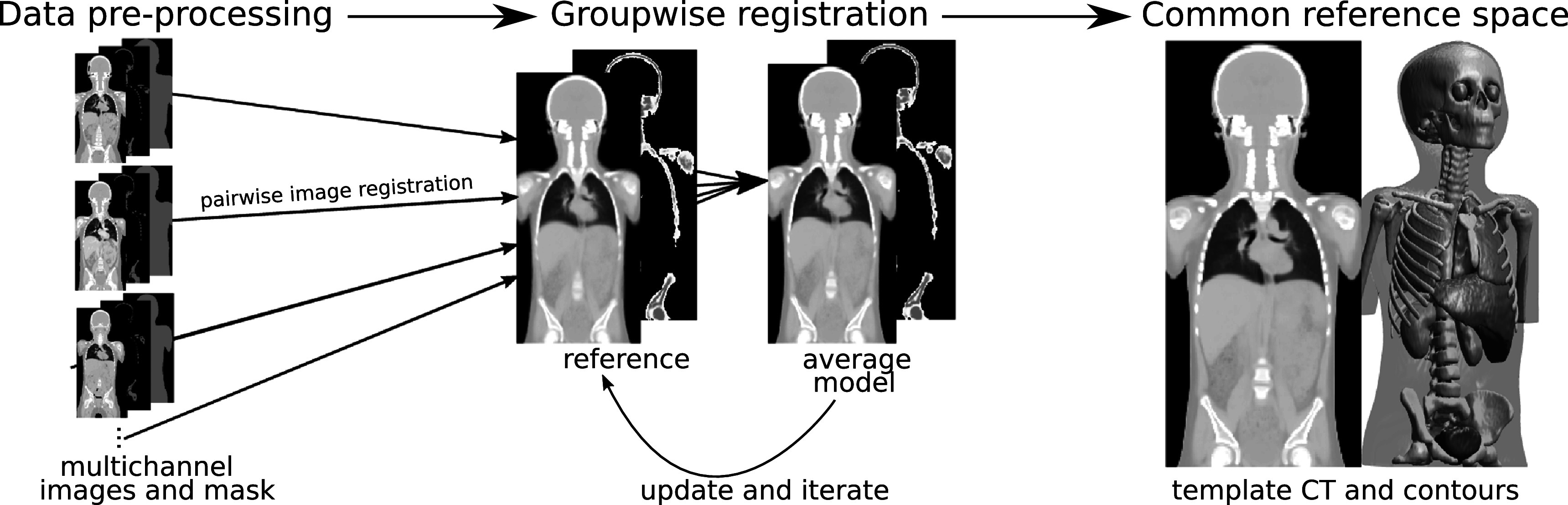
Schematic of pipeline used to generate the paediatric common reference space. The pipeline is divided into three main steps: (1) data pre-processing: definition of inputs to be used in pairwise registrations (multichannel images and corresponding masks); (2) groupwise image registration: iterative process of registering a set of images together to a common reference space; (3) Atlas construction: generate template CT and contours.

To spatially normalise complex anatomical and treatment imaging data, image registration is used to propagate information from individual subjects onto a common reference space. The first key step is to define a reference space representative of the population being studied. For our application, a simple, common, popular and scalable approach is to choose as reference space the planning computed tomography (CT) scan of a representative subject from the population (e.g. subject with average age or average height). However, the selection of a single subject as reference space introduces bias to the registrations which propagates to subsequent analysis. For example, if the selected reference volume has atypical anatomical features then all registrations are potentially more challenging and will estimate atypical and/or implausible transformations (Namburete *et al* 2018). Choosing an adequate reference is a challenging problem, particularly for the paediatric cancer population, known to be heterogeneous and prone to deviations in anatomy. Anatomical variations can occur simply due to changes with age, but more complex variations can occur with the treatments used, increasing the risk of individual subjects having atypical features. For example, some require invasive therapies which may cause co-morbidities and require additional interventions that are visible on CT imaging (such as the use of shunts or changed anatomy from surgical interventions). For such reasons, we opted to construct the reference space using groupwise image registration and paediatric radiotherapy CT images. Groupwise image registration is a process that iteratively alternates between co-registration of all subjects to a reference image and updating this reference image with the average model produced. Figure 1 provides a schematic overview of the pipeline proposed, which is detailed in the following sections.

#### 2.1.1. Patient data

The paediatric radiotherapy data used in our study was requested in line with the internal information governance procedures of the University College London Hospital (UCLH) Radiotherapy Department and was provided as fully-anonymised datasets. For atlas construction we used data from twenty children historically treated with 3D conformal photon CSI. This included 10 boys and 10 girls with a median age of 8 years (range: 3–15 years). All patients underwent a CT scan of the brain and whole spine, immobilised in the treatment position, for radiotherapy planning purposes. For simplicity, CSI CT scans are labelled as whole-body (as all major organs are visible), but limbs are usually partially out-of-field. Imaging resolution for all scans was 0.98 × 0.98 × 2.5 mm^3^. The following RT structures were used for validation purposes in the study: central nervous system (CNS) (whole brain and spinal canal down to L3), ocular globes, lungs, liver, kidneys and bladder. This set of contours was chosen to be clinically relevant and to cover different regions of the body. Clinically available contours were used if possible, and missing contours were additionally generated. Hence, the segmentation dataset had inter-user variability. All new contours were reviewed to clinically acceptable standards by an oncologist (EC) and/or physicist (CV). Manual segmentation and review was performed using the open-source software ITK-SNAP (Yushkevich *et al* 2006). Simple post-processing was employed before analysis to all contours to remove common segmentation errors (e.g. remove holes and discard small regions outside organ).

#### 2.1.2. Pre-processing

The following pre-processing steps were applied to all CTs prior to atlas construction to generate the inputs for image registration. The CTs were corrected to exclude external elements (e.g. couch and anaesthesia equipment) and to ignore shunts and high-intensity artefacts. External elements were defined as voxels outside the body contour and were overwritten as air (HU = −1000). High-intensity artefacts were replaced with ‘NaN’ value so that they would not contribute to the average image constructed at each iteration. Furthermore, since the location and volume of bowel gas is variable between patients and hence there is no true one-to-one mapping, bowel gas regions (defined from the body, inferior to the lungs, as HU < −200) were overwritten with water intensity (HU = 0). A binary mask was defined as a dilation of the body contour and used as input to speed the registrations.

Inter-subject image registration was particularly challenging in the CSI paediatric population due to the heterogeneous anatomy of children of both sexes aged 2–16 years. To guide the deformable registrations between subjects with different body sizes, the input images were defined as multichannel images. The first channel corresponded to the pre-processed CT image, while the second channel corresponded to the binary mask of the individual’s skeleton (defined as HU > 150). All multichannel CTs (and respective masks) were also automatically cropped in the anterior-posterior direction to further reduce computation time and memory requirements.

The binary images used in the pre-processing were generated semi-automatically based on thresholding of the CTs, morphological operations and existing manual RT segmentations, and then visually inspected and manually corrected (if needed) to remove gross labelling errors.

#### 2.1.3. Groupwise image registration

We constructed a reference space representative of the paediatric radiotherapy population using groupwise image registration. The output of interest was the final average intensity image produced, which we denominate as the ‘template CT’ from here onwards. We have modified the method available in NiftyReg (https://github.com/KCL-BMEIS/niftyreg) for groupwise registration, tailoring it for co-registration of whole-body CT images of paediatric subjects. The process was initialised by automatically selecting the initial reference to be the subject with closest age to the average age of the population. The pipeline then alternated between pairwise registration of all images to the reference image and updating the reference image at the end of each iteration. The updated reference was obtained by averaging the intensities of all the pairwise registration results whilst enforcing the mean of all transformations to be the identity transform. The complexity of the transformation model increased with iteration number, from rigid to fine deformable image registration (DIR). Coarser registrations allowed one to initially capture the large global variations in subject height and weight, which were followed by finer registrations to capture the smaller intra-patient variations in organ shape and size. This refinement process hence facilitated the co-registration of subjects with different body sizes and reduced computation times. A total of eleven iterations (one rigid-only, two affine and eight deformable) was empirically defined as further iterations were found to not provide sharper mean images.

Rigid and affine iterations used the block matching-based algorithm available in NiftyReg (Ourselin *et al* 2001, Modat *et al* 2014). Multichannel and cropped images were generated at the end of the last affine step and used in the following iterations. The DIR steps made use of the velocity fields parametrisation of the B-spline free form deformation based algorithm (Rueckert *et al* 1999, Modat *et al* 2012), guaranteeing transformations that were diffeomorphic, symmetric and inverse-consistent. All pairwise deformable registrations used a multi-resolution approach with five levels. Locally normalised cross correlation and sum squared difference were used as similarity metrics in the CT and skeleton channels, respectively. Bending energy and linear elasticity penalty terms were used for regularisation in all registrations to encourage smooth deformations. The number of levels to perform and control point spacing were updated with iteration number to progressively recover finer deformations. The finest registrations were carried out using five resolution levels and a control grid spacing of 12.5 mm.

In addition to generating the average CT image, at the last iteration the corresponding average contours were also generated by propagating and averaging the organ contours for all subjects (majority voting), using the same transformations.

### 2.2. Evaluation experiments

To evaluate the constructed paediatric atlas for spatial normalisation, an independent set of 53 patients historically treated at UCLH was used. This evaluation dataset included 31 boys and 22 girls with a median age of 5 years (range: 1–16 years) from a variety of disease cohorts, including CSI (*n* = 30), abdominal neuroblastomas (*n* = 18), brain tumours (*n* = 3) and Ewing sarcomas (*n* = 2). Similarly to the data described in section 2.1.1, planning CT images and corresponding contours (CNS, ocular globes, lungs, liver, kidneys and bladder) were used for analysis. Clinical RT doses were also available for every subject, with prescription and fractionation varying between patients. All subjects were registered to the template CT using affine followed by non-rigid registration. The registration parameters and pre-processing strategy were similar to those used for the finest pairwise registrations in the groupwise pipeline.

The quality of the spatial normalisation process was evaluated considering how well the registrations aligned organs at the common reference space and if dose-volume properties were preserved after registration; furthermore, smooth deformations were important to promote the preservation of dose-volume characteristics. To evaluate the mapping in regions without manual labels, intensity-based similarity and deformation metrics were calculated. All metrics of image quality, contour similarity and dose-volume characteristics used were defined in table 1 and briefly described in the following paragraphs. The following nomenclature was used to differentiate the different spaces where three-dimensional subject-specific information (i.e. CT, contours and dose) were defined: *s* = information of each subject in its native coordinate system; *t* = information of the template CT itself on its own coordinate system (common reference space); $s\to t$ = information propagated from the subject space into the template CT coordinate system via image registration.

**Table 1.** Quantities used in the evaluation of the spatial normalisation.

Quantity	Equation	Description
Normalised cross-correlation (${\mathrm{NCC}}$)	${\mathrm{NCC}}=\tfrac{\displaystyle \sum \left[\left({{I}}_{{s}\to {t}}\left({r}\right)-\,\overline{{{I}}_{{s}\to {t}}}\right)\times \left({{I}}_{{t}}({r})-\overline{{{I}}_{{t}}}\right)\right]}{\sqrt{\displaystyle \sum {\left({{I}}_{{s}\to {t}}\left({r}\right)-\,\overline{{{I}}_{{s}\to {t}}}\right)}^{2}\times \displaystyle \sum {\left({{I}}_{{t}}\left({r}\right)-\overline{{{I}}_{{t}}}\right)}^{2}}}$	${{I}}_{{A}}({r})$ is the pixel intensity (HU) in image *A* at voxel *r*, and $\overline{{{I}}_{{A}}}$ is the mean intensity. $\mathrm{NCC}$ is a metric of the degree of similarity between images. Ranges from −1 to 1 with higher values representing higher image similarity
Root mean square error of intensities (${{\mathrm{HU}}}_{{\mathrm{rms}}}$)	${{\mathrm{\Delta }}{\mathrm{HU}}}_{{\mathrm{rms}}}=\sqrt{\tfrac{1}{n}\sum {\left({I}_{s\to t}\left(r\right)-{I}_{t}(r)\right)}^{2}}$	${{\mathrm{\Delta }}{\mathrm{HU}}}_{{\mathrm{rms}}}\,\,$provides a measure of disparity in image intensities. Units of ${{\mathrm{\Delta }}{\mathrm{HU}}}_{{\mathrm{rms}}}:$ HU
Average absolute local volume change (${{\mathrm{LVC}}}_{{\mathrm{avg}}}$)	${{\mathrm{LVC}}}_{{\mathrm{avg}}}=\tfrac{1}{n}\displaystyle {\sum }_{}\left|{\mathrm{LVC}}\left(r\right)\right|\,,$	${\mathrm{LVC}}$ is the local volume change, computed from the determinant of the Jacobian (${J}$) of the deformation (Pilia *et al* 2019)
	where	
	${\mathrm{LVC}}({r})=\left\{\begin{array}{c}1-1/{J}({r})\\ {J}\left({r}\right)-1\end{array}\right.\,\begin{array}{c}{J}\left({r}\right)< 1\\ {J}({r})\geqslant 1\end{array}$	
Dice similarity coefficient (DSC)	${\mathrm{DSC}}=2\times \displaystyle \frac{{{V}}_{{t}}{\cap }^{\,}{{V}}_{{s}\to {t}}\,}{\left|{{V}}_{{t}}\right|+\left|{{V}}_{{s}\to {t}}\right|}$	${{V}}_{{A}}$ represents the voxels that define a volume of interest *A*. DSC ranges from 0 to 1, with higher values representing better contour overlap
Jaccard coefficient (JC)	${\mathrm{JC}}=\tfrac{{{V}}_{{t}}{\cap }^{\,}{{V}}_{{s}\to {t}}\,}{{{V}}_{{t}}{\cup }^{\,}{{V}}_{{s}\to {t}}\,}$	JC ranges from 0 to 1, with higher values representing better contour overlap
Average distance between surfaces (${{\mathrm{DT}}}_{{\mathrm{avg}}}$)	${{\mathrm{DT}}}_{{\mathrm{avg}}}=\,{\mathrm{\max }}\{\,\overline{{{\mathrm{DT}}}_{{s},\,{s}\to {t}}},\,\overline{{{\mathrm{DT}}}_{{s}\to {t},\,{s}}}\}$	$\overline{{{\mathrm{DT}}}_{{A},\,B}}$ is the mean of the distribution of values for the distance between each point on the surface of volume *A* to the closest point on the surface of volume *B.* Units of ${{\mathrm{DT}}}_{{\mathrm{avg}}}:$ mm
Distance between centroids (${\mathrm{\Delta }}\mathrm{TR}$)	${\mathrm{\Delta }}\mathrm{TR}=\parallel {{R}}_{{s}\to {t}}-{{R}}_{{t}}\parallel \,$	${{R}}_{{A}}$ is the centroid of a volume *A*. Units of ${\mathrm{\Delta }}\mathrm{TR}:$ mm
Relative difference of areas of DVHs (${\mathrm{RDA}}$)	${\mathrm{RDA}}=\tfrac{\displaystyle \int \left|{{\mathrm{DVH}}}_{{s}\to {t}}-{{\mathrm{DVH}}}_{{s}}\right|{dx}}{{\mathrm{\max }}\left\{\displaystyle \int {{\mathrm{DVH}}}_{{s}}{dx},\displaystyle \int {{\mathrm{DVH}}}_{{s}\to {t}}{dx}\right\}}$	${{\mathrm{DVH}}}_{{A}}$ is dose-volume histogram for volume *A.* ${\mathrm{RDA}}$ ranges from 0 and 1, with lower values representing better DVH preservation. (Adapted from Acosta *et al* 2013)
Dose-organ overlap (${\mathrm{DOO}}$)	${\mathrm{DOO}}=\tfrac{\displaystyle {\int }_{{{V}}_{{t}}\cap {{V}}_{{s}\to {t}}}{{D}}_{{s}\to {t}}\left({x}\right){dx}}{\displaystyle {\int }_{{{V}}_{{t}}\cup {{V}}_{{s}\to {t}}}{{D}}_{{s}\to {t}}\left({x}\right){dx}}$	${{D}}_{{A}}$ is the three-dimensional dose matrix inside volume *A.* ${\mathrm{DOO}}$ ranges from 0 and 1, with higher values representing better DVH preservation (Acosta *et al* 2013)

Note. Definitions: • *s* = information of each subject in its native coordinate system; • *t* = information of the template CT itself on its own coordinate system (common reference space); • ${s}\to t\,$ = information propagated from the subject space into the template CT coordinate system.

Intensity-based similarity was assessed by calculating the normalised cross correlation (${\mathrm{NCC}}$) and the root mean square error (${{\mathrm{HU}}}_{{\mathrm{rms}}}$) between the deformed CTs and template CT. To demonstrate the range of deformations recovered, we also computed the average absolute local volume change (${{\mathrm{LVC}}}_{{\mathrm{avg}}}$) using the determinant of the Jacobian of the pairwise deformations (Pilia *et al* 2019).

To describe the similarity between contours defined in the template space (${V}_{t}$) and the equivalent contours propagated from each subject to this space via image registration (${V}_{s\to t}$), we computed the dice similarity coefficient (${\mathrm{DSC}}$), Jaccard coefficient (${\mathrm{JC}}$), average distance between surfaces (${{\mathrm{DT}}}_{{\mathrm{avg}}}$) (Mishchenko 2015) and distance between centroids (${\mathrm{\Delta }}\mathrm{TR}$). These quantities measure accuracy of the registrations in mapping organ volume, location and shape.

Spatial normalisation should preserve the dose-volume properties of each individual subject, such that DVH-based models of side-effects would be similar if performed on the subject or common reference space. The differences in the DVHs computed in the subject (${{\mathrm{DVH}}}_{{s}}$) and template (${{\mathrm{DVH}}}_{{s}\to {t}}$) spaces were assessed using the relative difference of areas of DVHs (${\mathrm{RDA}}$) and dose-organ overlap (${\mathrm{DOO}}$) (Acosta *et al* 2013).

It should be noted that not all patients included had planning CTs that covered the same field-of-view, which may impact in the metrics reported. When computing different measures of registration quality, pixels outside the body and the common field-of-view were excluded from analysis.

### 2.3. Critical evaluation of spatial normalisation for radiation-induced second malignant neoplasms (SMNs) risk

**Figure 2.**
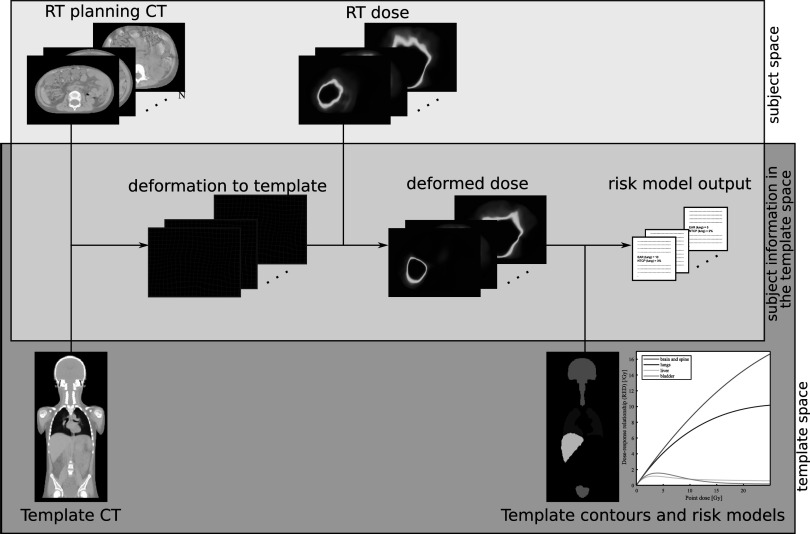
Diagram showing data workflow for spatial normalisation to facilitate radiation-induced late effects research.

Our aim in this part of the study was to demonstrate the potential of the proposed atlas-based approach to facilitate radiation-induced late effects research in childhood cancer treatment (figure 2). The risks of radiation-induced SMNs were estimated for a group of patients using the common reference space ($s\to t$) and compared the equivalent values using the original subject space (*s*). For this purpose, we used a subset of subjects for whom clinically acceptable dual radiotherapy plans were available. This included fourteen patients from different disease cohorts (from the *n* = 53 evaluation cohort): CSI (*n* = 3), abdominal neuroblastomas (*n* = 7), brain tumours (*n* = 3) and Ewing’s sarcoma (*n* = 1). A photon plan and a pencil-beam scanning proton therapy plan were available for risk estimation for each subject. As different patient groups and treatment modalities were included, this subgroup had variability in the 3D dose distributions considered. This was intentionally chosen such that organs were located in both homogeneous dose regions and within dose gradients, and with varying position relative to the RT field (i.e. inside the RT target, near-target and out-of-field). In the case of the proton therapy plans, an estimation of homogeneous whole body neutron dose was included (Schneider *et al* 2002). In addition to assessing mean and maximum organ doses (${{D}}_{{\mathrm{avg}}}$ and ${{D}}_{{\mathrm{\max }}}$), (i.e. linear dose-response model), a mechanistic model was used to estimate the excess absolute risk (${\mathrm{EAR}}$) of radiation-induced carcinomas in the CNS, lungs, liver, and bladder (Schneider *et al* 2011). This model accounts for cell killing, repopulation and fractionation effects and was developed for therapeutic exposures. ${\mathrm{EAR}}$ was estimated from the dose to volume data using the mechanistic dose-response model (i.e. nonlinear model), and an age-dependent modifying function. Parameters depend on the tissues being irradiated, and are available in the original publication (Schneider *et al* 2011). We reported the ${{D}}_{{\mathrm{avg}}},$ ${{D}}_{{\mathrm{\max }}},$ and ${\mathrm{EAR}}$ for both modalities, as well as the risk ratio (${\mathrm{RR}}$) between modalities. For convenience, the RR was defined to range between 0 and 1 such that it does not depend on which modality is estimated as superior:

\begin{eqnarray*}{\mathrm{RR}}=\,{\mathrm{\min }}\left\{\displaystyle \frac{{{\mathrm{EAR}}}_{{\mathrm{protons}}}}{{{\mathrm{EAR}}}_{{\mathrm{photons}}}},\displaystyle \frac{{{\mathrm{EAR}}}_{{\mathrm{photons}}}}{{{\mathrm{EAR}}}_{{\mathrm{protons}}}}\right\}.\end{eqnarray*}

### 2.4. Implementation details and data analysis

The pipelines for atlas construction, subject propagation, validation workflow and SMN models were implemented in Matlab 2019a (Mathworks Inc.). Statistical analysis was also performed in Matlab, using the Statistical Toolbox, with statistical significance set at 5%. Not every patient included had complete segmentation sets due to variations in the field-of-view covered by the planning CT. Therefore, the dimensions of the samples used were variable.

## 3. Results

### 3.1. Overview of the template space

**Figure 3.**
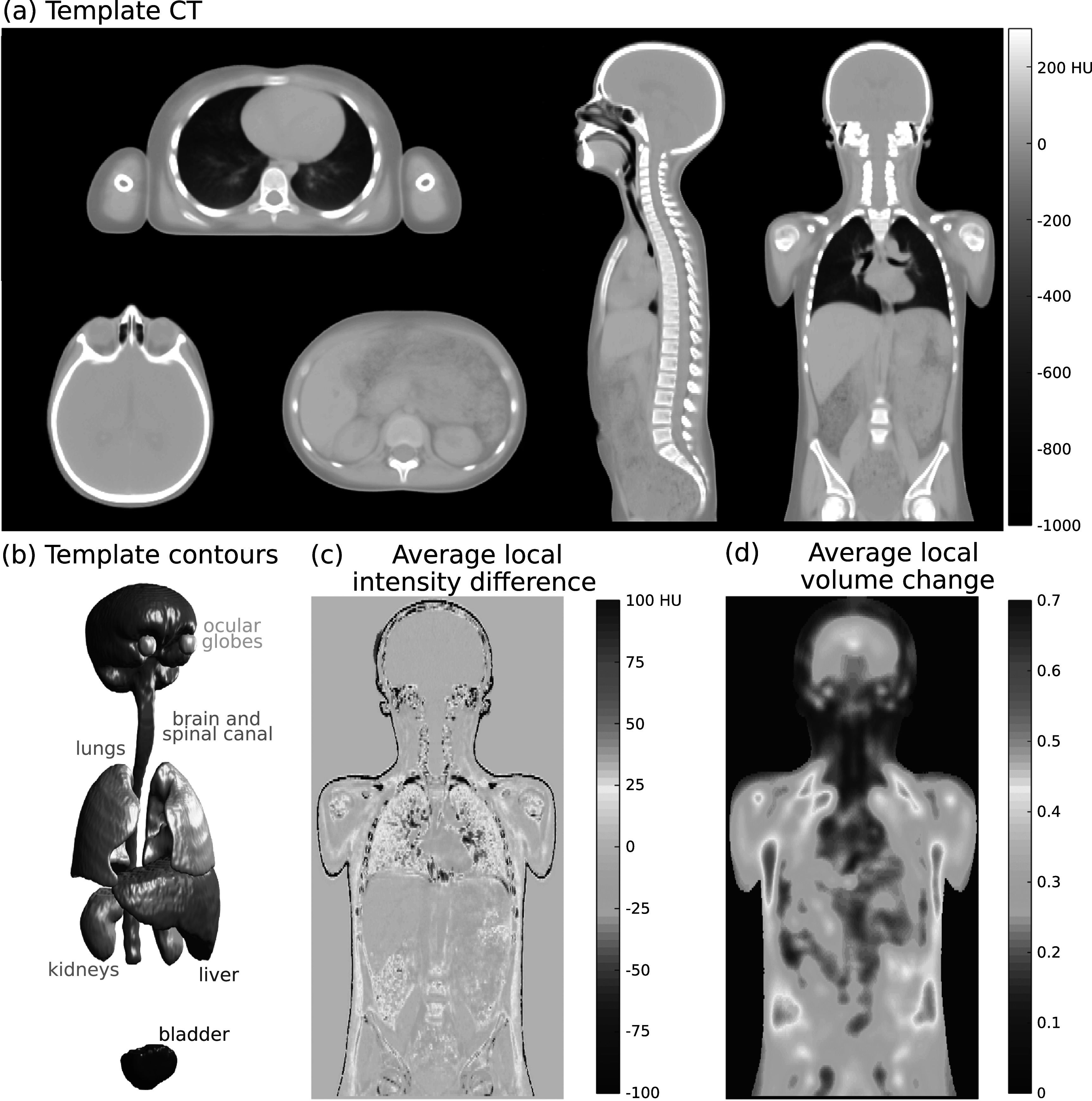
(a) Template CT generated using groupwise image registration on selected axial, sagittal and coronal views and (b) corresponding contours. (c) Map of local average intensity difference between template and subject CTs. (d) Map of average absolute value of the local volume change when co-registering subjects. Maps resulted from averaging over the twenty subjects used for atlas construction, excluding pixels away from the body.

The atlas construction took approximately 35.5 ± 0.5 h on a dual Intel^®^ Xeon^®^ Gold 6134 CPU (3.20 GHz), 128 GB memory (computation was repeated three times). Figure 3 shows the atlas constructed using the twenty CSI subjects, as well as segmentations, average HU and volume differences per voxel. The deformable registration of all subjects (n = 73) to this template took 65 ± 30 min (per subject) on the same system.

**Figure 4.**
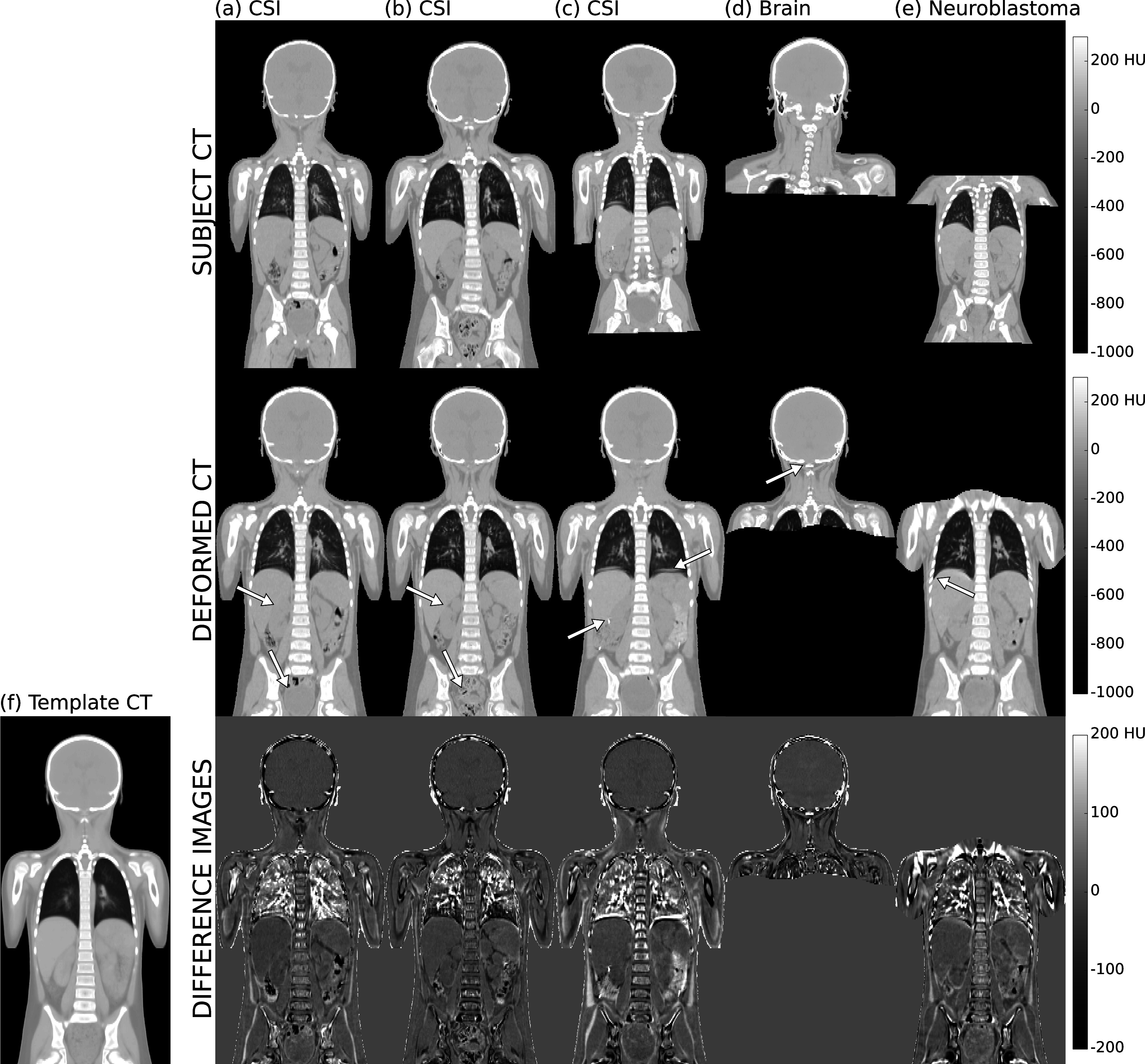
Examples of registrations to the (f) template CT, including subjects from (a)–(c) craniospinal irradiation (CSI) and (d)–(e) other disease cohorts. Top row: subject CT scans (rigid-only alignment). Middle row: deformed CT scans. Bottom row: difference map between registered scans (deformable registration) and template CT. In general, the registrations can successfully align the anatomy, but specific challenges arise in different body regions. Contrast in anatomical boundaries is fundamental to guide the registration, but clinical scan quality may vary. The boundary between liver and kidneys is less sharp in (a) than in (b), for example (top arrows), leading to poorer anatomical matching in this region; likewise, large deformations of the bladder are challenging to recover, where filling varies from (a) full to (b) empty (bottom arrows). Imaging artefacts can also be problematic, such as (c) motion artefacts (top arrow) and/or the use of contrast agents (bottom arrow). Note that high-intensity artefacts were masked out during the registration. Different disease cohorts have differences in image acquisition of parameters, patient positioning and imaged field-of-view ((d) brain tumour versus (e) abdominal neuroblastoma). Arrows indicate examples of misregistration regions on the skeleton (d) and soft tissues (e).

Figure 4 shows some examples of registrations for different disease cohorts, highlighting some of the common pitfalls of the spatial normalisation process. The registrations were able to successfully align the overall anatomy at the common reference space despite the wide variation in age, height, and weight between subjects. Large local deformations were challenging to completely capture, and visually we could identify common patterns of misregistration such as local misalignment of individual bones (e.g. individual vertebrae and ribs) and poor matching at soft tissue boundaries (e.g. between right kidney and liver). Image quality was an important source of registration variability contributing to fuzzier aspect in regions without consistently sharp anatomical boundaries.

The average values for ${\mathrm{NCC}}$ was 0.97 ± 0.01, indicating a good match between deformed and template CTs. ${{\mathrm{HU}}}_{{\mathrm{rms}}}$ was 94 ± 10 HU, which also indicates good global alignment. A level of difference in intensity was expected due to the variability in CT intensities between patients and the fuzzier aspect of the template CT. We also report a ${{\mathrm{LVC}}}_{{\mathrm{avg}}}$ of 0.38 ± 0.16, which is indicative of the magnitude of volume changes that must be captured by the registrations, with the largest values being attributed to variations in patient size across the population studied.

### 3.2. Evaluation of anatomical and dose mapping

**Figure 5.**
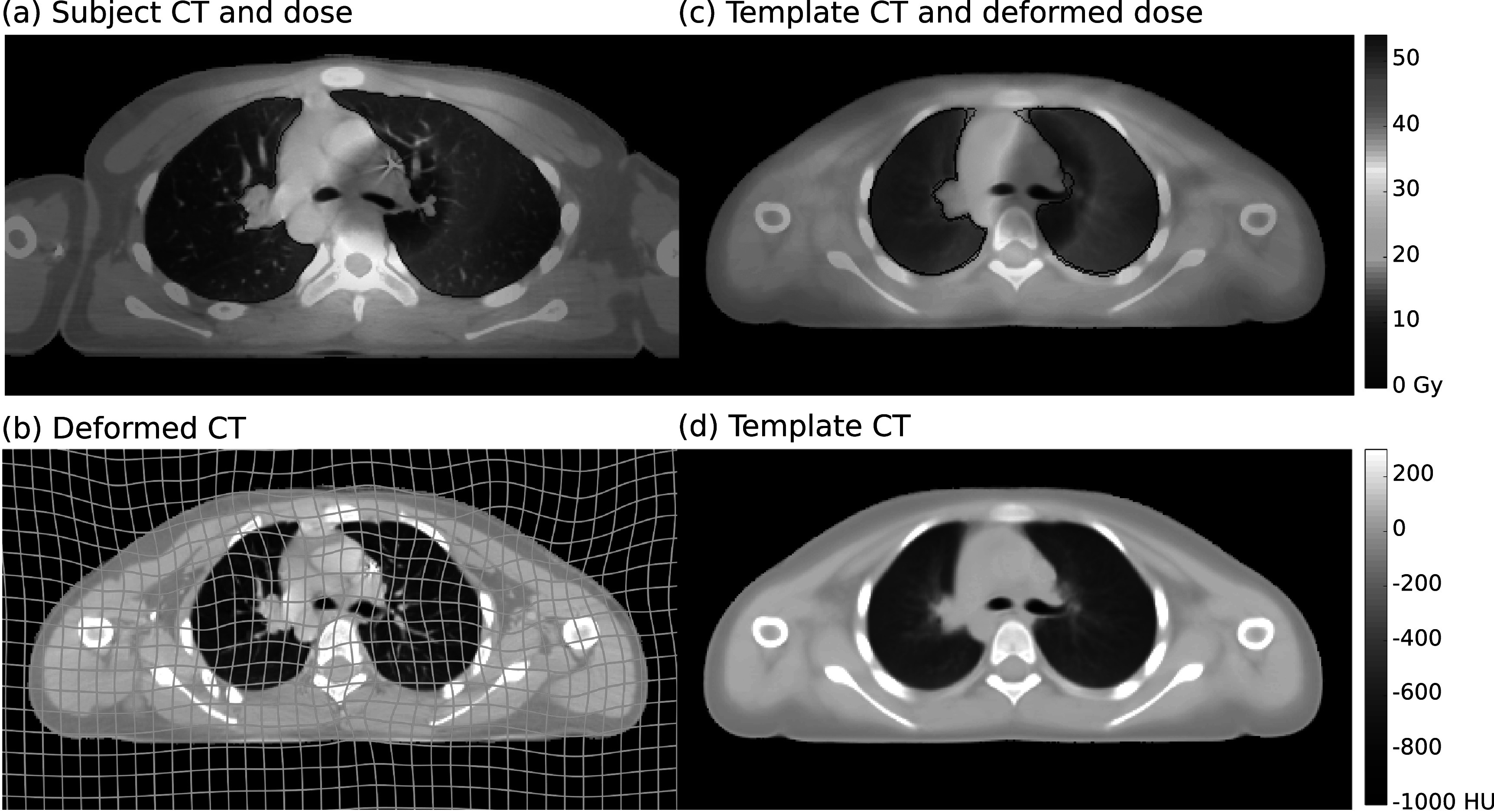
Example of data (images, contours and doses) used in the evaluation experiments. (a) CT, lungs contour (red) and dose distribution for one of the subjects included in the analysis (Ewing sarcoma); (b) subject CT propagated to the template (affine followed by deformable registration), and corresponding deformation grid; (c) template CT and lungs contour (blue), and overlaid deformed lungs contour (red) and deformed dose distribution; (d) template CT by itself.

Figure 5 shows an example of the different CTs, contours and doses used in the evaluation of the anatomical and dose mapping.

**Figure 6.**
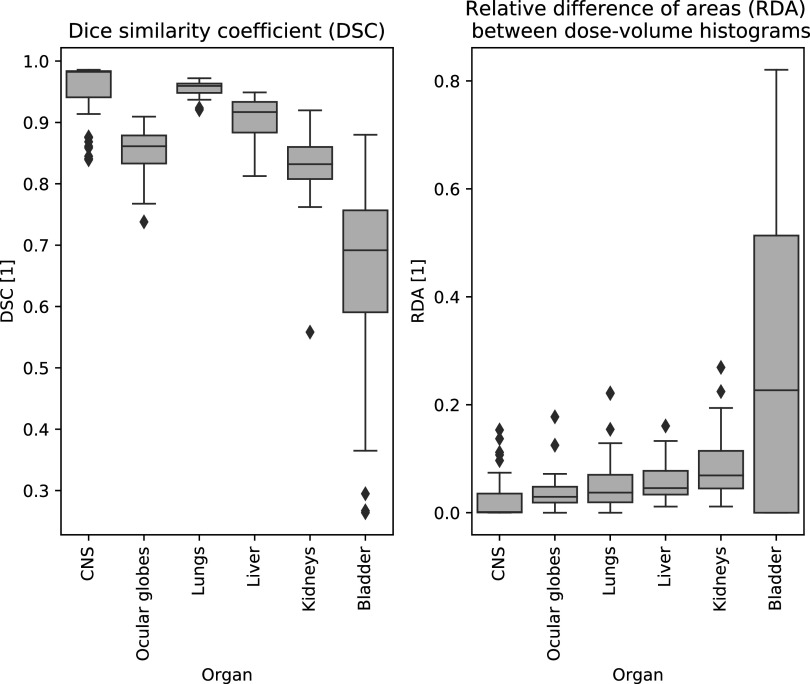
Boxplots for distribution of dice similarity coefficient (DSC) and relative difference of areas (RDA) after spatial normalisation. Subjects with contours defined available for analysis: *N* = {53, 34, 52, 49, 49, 49} for {CNS, Ocular Globes, Lungs, Liver, Kidneys, Bladder}. Outliers fall outside the ±2.7 standard deviation range.

The quantities calculated for each organ are summarised in table 2. Details of the distribution in volume and dose per organ within the patient group are also provided. Figure 6 complements this information by displaying the distribution of the ${\mathrm{DSC}}$ and ${\mathrm{RDA}}$ for all contours, chosen as representative measures of anatomical and dose mapping results.

**Table 2.** Quantitative evaluation of contour and dose deformation for different organs, expressed as mean ± standard deviation.

	Volume of interest					
Quantity	CNS	Ocular globes	Lungs	Liver	Kidneys	Bladder
DSC	0.95 ± 0.05	0.85 ± 0.04	0.96 ± 0.01	0.91 ± 0.03	0.83 ± 0.06	0.65 ± 0.16
JC	0.92 ± 0.08	0.74 ± 0.06	0.92 ± 0.02	0.83 ± 0.5	0.71 ± 0.08	0.50 ± 0.16
${{\mathrm{DT}}}_{{\mathrm{avg}}}$ (mm)	0.7 ± 1.0	0.9 ± 0.4	0.8 ± 0.3	2.7 ± 1.1	2.5 ± 1.3	7.1 ± 3.8
${\mathrm{\Delta }}\mathrm{TR}$ (mm)	2.9 ± 5.7	1.7 ± 0.8^a^	1.3 ± 0.7^a^	4.9 ± 2.7	7.0 ± 4.5^a^	9.6 ± 4.6
${\mathrm{RDA}}$	0.02 ± 0.04	0.04 ± 0.04	0.05 ± 0.05	0.06 ± 0.03	0.09 ± 0.06	0.29 ± 0.28
${\mathrm{DOO}}$	0.92 ± 0.07	0.75 ± 0.07	0.86 ± 0.05	0.81 ± 0.06	0.65 ± 0.09	0.36 ± 0.18
Number of subjects in analysis (no, %)	53	34	52	49	49	49
	(100%)	(64%)	(98%)	(92%)	(92%)	(92%)
Volume (ml), median (range)	1331	16	597	645	121	64
	(29–1891)	(10–21)	(49–2834	(328–1632)	(62–284)	(10–350)
Mean organ dose (Gy), median (range)	23.6	26.2	3.5	6.7	5.2	1.1
	(1.3–38.9)	(0.2–35.9)	(0–16.4)	(1.1–25.0)	(1.4–14.1)	(0–16.3)

Note. DSC = dice similarity coefficient JC = Jaccard coefficient ${{\mathrm{DT}}}_{{\mathrm{avg}}}$ = average distance between surfaces ${\mathrm{\Delta }}\mathrm{TR}$ = distance between centroids ${\mathrm{RDA}}$ = relative difference of areas of DVHs ${\mathrm{DOO}}$ = dose-organ overlap CNS = brain and spinal canal.

^a^ For organs composed of two separate volumes, ${\mathrm{\Delta }}\mathrm{TR}$ reported is the maximum value of the individual sub-volumes.

In general, better matching was found for organs in the head and thorax, than for those located in the abdomen and pelvis. The poorest performance was achieved in the bladder, the organ that exhibited the largest inter-subject volume variation due to differences in filling (excluding cases where other organs were partially imaged). Upon visual inspection of outliers for other regions of interest, the worst performances corresponded to abnormal anatomies, such as individuals with enlarged kidneys or inflammation in the lungs. A common error (affecting approximately 1 in 5 subjects) in CNS registration corresponded to misregistration of individual spinal vertebrae, which could lead to mismatch of the inferior end of the spinal canal (which was consistently defined at L3 for all subjects and template). Registrations also struggled with the kidney superior/inferior boundaries where contrast is poor, reflected in higher errors mapping the structure centroid.

Registration quality was generally better for CSI than for other cohorts (abdominal neuroblastoma, brain and Ewing’s sarcoma). For example, the average DSC for the liver was 0.92 ± 0.02 and 0.88 ± 0.03 for CSI and other cohorts, respectively.

Considering CSI subjects only, we also investigated the relationship between all metrics and absolute age difference (relative to the template CT). We did not find any strong evidence that subjects with ages furthest away from average were better registered to the template CT. When pooling data for all organs and subjects, the Pearson’s correlation coefficients were −0.05, −0.04, 0.08, 0.04, 0.04 and −0.04 (*p* > 0.05) for DSC, JC, ${{\mathrm{DT}}}_{{\mathrm{avg}}},$ ${\mathrm{\Delta }}\mathrm{TR},$ ${\mathrm{RDA}}$ and ${\mathrm{DOO}},$ respectively, showing a weak correlation between age similarity and quality of anatomical and dose deformation to the template CT.

### 3.3. Additional evaluation experiments

Separately to the previous experiments, we also evaluated anatomical and dose mapping to the template for the twenty subjects used in the atlas construction. We found no strong statistical evidence that the measured quantities were better for CSI subjects used for atlas construction than for those used only for evaluation (*p* > 0.05, Wilcoxon rank sum test).

**Figure 7.**
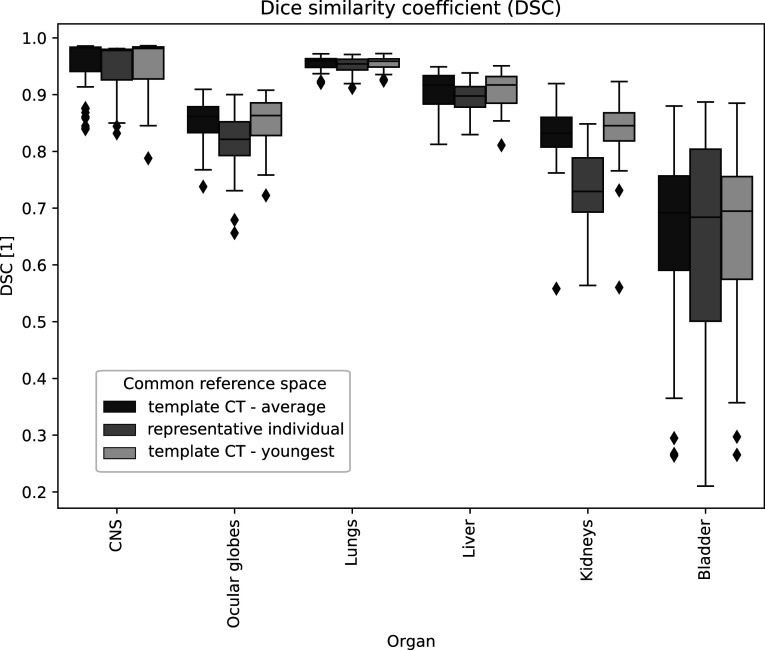
Boxplots for distribution of dice similarity coefficient (DSC) after spatial normalisation comparing different common reference spaces: proposed template CT, representative population individual, and templates CT generated with a different initial template (youngest subject). Subjects with contours defined available for analysis: *N* = {53, 34, 52, 49, 49, 49} for {CNS, Ocular Globes, Lungs, Liver, Kidneys, Bladder}. Outliers fall outside the ±2.7 standard deviation range. Note how similar results are achieved for both template CTs, with higher metrics found relative to the representative subject.

The anatomical and dose mapping achieved with other choices of common reference space was also assessed, and compared to the proposed template CT. First, we calculated all metrics when spatially normalising to a population-representative individual. The chosen individual was the subject closest to the average age, also used to initiate the atlas construction. Then, we repeated the same analysis but choosing as reference the youngest and oldest subjects (proxy for least representative subjects) to highlight the importance of adequate reference selection for spatial normalisation. Results are shown in table 3 when pooling data for all organs and subjects. Figure 7 showcases the differences in DSC for all organs when comparing spatial normalisation to the template CT and representative subject only. Spatial normalisation to a representative subject generally resulted in poorer metrics achieved in comparison to the template CT but improved against using as reference space subjects with more dissimilar ages (and hence expected to be less representative of the population).

**Table 3.** Quantitative evaluation of contour and dose deformation to different common reference spaces: proposed template CT (evaluated in sections 3.1 and 3.2), population individuals (youngest, average and oldest in age), and template CTs generated with different initial reference (youngest and oldest subjects). Data pooled for all organs and subjects. Expressed as mean ± standard deviation. Note how similar results are achieved for all template CTs; representative subject is associated with poorer metrics but outperforms the less representative individuals.

		Other common reference spaces				
		Individuals			Template CT with different initialisation	
Quantity	Template CT	Average	Youngest	Oldest	Youngest	Oldest
DSC	0.86 ± 0.13	0.83 ± 0.15	0.75 ± 0.23	0.81 ± 0.14	0.86 ± 0.13	0.86 ± 0.13
JC	0.77 ± 0.17	0.74 ± 0.19	0.64 ± 0.26	0.70 ± 0.18	0.78 ± 0.17	0.78 ± 0.17
${{\mathrm{DT}}}_{{\mathrm{avg}}}$ (mm)	2.5 ± 2.8	2.7 ± 3.1	3.9 ± 3.8	4.6 ± 4.3	2.5 ± 2.8	2.4 ± 2.9
${\mathrm{\Delta }}\mathrm{TR}$ (mm)	4.6 ± 4.8	5.6 ± 6.0	8.4 ± 7.1	9.4 ± 8.2	4.6 ± 4.8	4.5 ± 4.8
${\mathrm{RDA}}$	0.09 ± 0.15	0.11 ± 0.17	0.12 ± 0.17	0.10 ± 0.13	0.10 ± 0.15	0.10 ± 0.15
${\mathrm{DOO}}$	0.73 ± 0.21	0.69 ± 0.07	0.62 ± 0.27	0.66 ± 0.21	0.73 ± 0.21	0.73 ± 0.21

Finally, to evaluate the impact of initial reference selection in the atlas constructed, the atlas construction process was repeated by iterating the initial reference selected over the remaining nineteen subjects. All atlases generated were comparable to the proposed atlas after affine registration (${\mathrm{NCC}}$ = 0.993 ± 0.003 and ${{\mathrm{HU}}}_{{\mathrm{rms}}}$ = 45 ± 9 HU), irrespective of which subject was used to initiate the process. Visually, the inter-atlas anatomical variability was small in comparison with the inter-subject variability presented in the patient group. Furthermore, all anatomical and dose mapping metrics were recalculated on two (out of nineteen) of these comparable atlases, the ones constructed using the youngest and oldest subjects as the initial reference. Similar metrics were found on the three different template CTs analysed, demonstrating the robustness of the atlas construction process. These results are also summarised in table 3 and figure 7.

### 3.4. Evaluation of a common reference space to facilitate the study of radiation-induced SMNs

**Figure 8.**
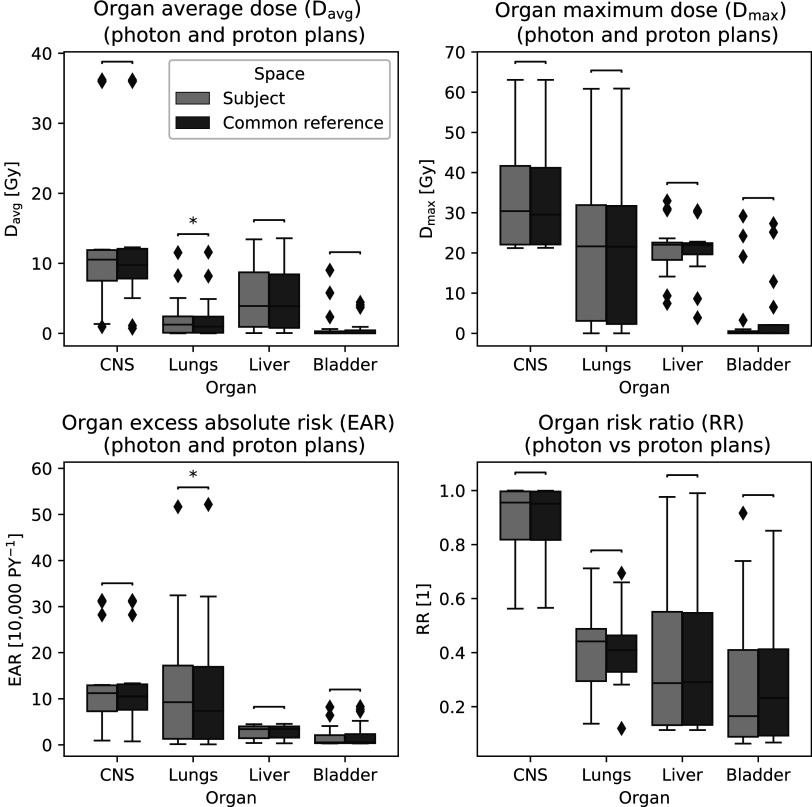
(a) Average dose (${D}_{{\mathrm{a}}{\mathrm{v}}{\mathrm{g}}}$), (b) maximum dose (${D}_{{\mathrm{m}}{\mathrm{a}}{\mathrm{x}}}$) and (c) excess absolute risk (EAR) calculated for CNS, lungs, liver and bladder considering both photon and proton therapy plans, and corresponding (d) risk ratio (RR) between the two modalities calculated using the dose onto the template CT and contours (subject to common reference space), and by using the dose on the native CTs and contours (subject space). Subjects with contours defined available for analysis: *N* = {14, 13, 10, 10} for {CNS, Lungs, Liver, Bladder}. Outliers fall outside the ±2.7 standard deviation range. Asterisks indicate *p* < 0.05 for Wilcoxon paired signed rank tests.

The radiation-induced SMN risk from photon and proton therapy treatments was estimated by propagating the dose onto the template CT and contours (subject to common reference space), and by using the dose on the native CTs and contours (subject space) (figure 8). There was no strong evidence of statistically significant differences in the ${{D}}_{{\mathrm{avg}}},$ ${{D}}_{{\mathrm{\max }}},$ ${\mathrm{EAR}}$ and ${\mathrm{RR}}$ calculated on the two spaces (Wilcoxon paired signed rank tests, *p*-values > 0.05 for the majority of data pairs). This suggests that analysis on the common reference or subject space is equivalent for DVH-based studies and the added uncertainties to dose-volume characteristics associated with the spatial normalistion have a small impact on the population level.

**Figure 9.**
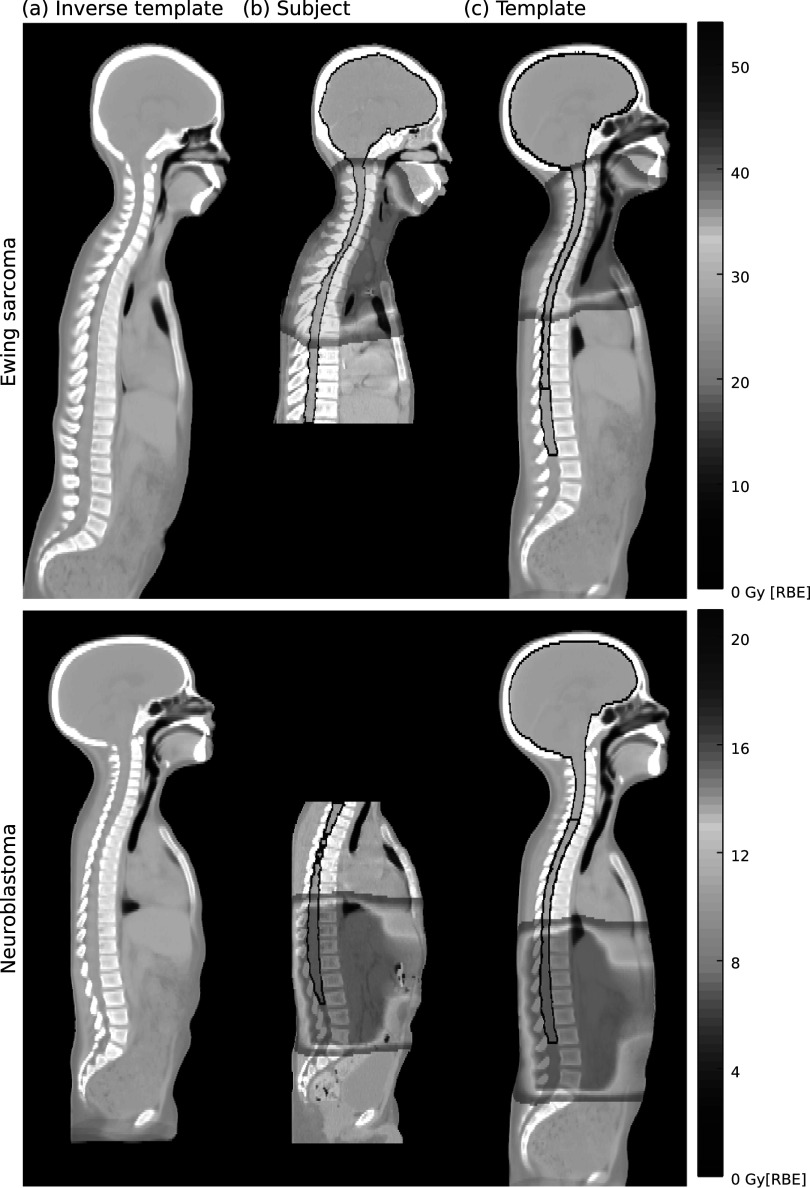
Two clinical cases demonstrating the advantages of dose analysis on the template CT (top: Ewing sarcoma; bottom: neuroblastoma). Propagating dose from subject space (b) to the common reference space (c) allows to explore the spatial relationships between dose and clinical end-points—for example, both cases irradiate comparable spinal volumes with 20 Gy or more, but at distinct anatomical regions. Furthermore, the template contour (blue) allows for DVH-based analysis in the absence of patient-specific segmentations (red) and to account for volume effects when only partial volumes were imaged. Finally, the template CT may be used to generate virtual phantoms to estimate out-of-field doses by, for example, using the patient-specific inverse deformations (a).

In figure 9 we showcase examples of proton plans for paediatric Ewing sarcoma and neuroblastoma to highlight the potential benefits of using the common reference space for SMN risk modelling and analysis. While prescription is very different between cohorts, both treatments irradiate the spine at similar dose levels (estimated *V*_20 Gy_ to the CNS of 3.1% and 2.3%, respectively) but at distinct sub-regions. Propagating dose to a common template space allows to explore how such spatial relationships may impact the relationships between dose and clinical end-points. Furthermore, the template CT and its contours (blue) allow for DVH-based analysis in the absence of segmentations on the subject CT (red) and to account for volume effects when only partial volumes were imaged. For example, in the neuroblastoma case the absolute EAR for the CNS would be 12.9 per 10 000 person-years if not properly accounting for partial imaging of this organ (i.e. brain is outside the imaged region); 0.54 per 10 000 person-years is the estimated absolute value in the template space. Note that when calculating EAR shown in figure 8, we only used the common field-of-view between subject and template for a fairer comparison. Finally, the template CT may be used to generate virtual phantoms to estimate out-of-field doses. A possible way of doing this is by using the patient-specific inverse deformations to generate a patient-specific phantom.

## 4. Discussion

In this study, we investigated the feasibility of atlas-construction and spatial normalisation to facilitate voxel-based analysis of radiation-induced toxicities in paediatric radiotherapy patients. This methodology exploits imaging and contour segmentation information from a CSI cohort to spatially standardise the heterogeneous paediatric population and facilitate subsequent analysis. The methodology was applied to paediatric CSI, abdominal neuroblastoma, Ewing sarcoma and brain tumour patients. The single synthetic template generated was able to satisfactorily account for considerable variability in age and gender. This demonstrates the potential of spatial normalisation of a heterogeneous population to facilitate subsequent analysis of varied clinical end-points in larger paediatric populations. To the best of our knowledge, this is the first-time atlas construction and spatial normalisation were investigated for whole-body images of paediatric cancer patients who underwent radiotherapy.

We chose for atlas construction the CSI patient group for its potential as a reference frame, as the radiation fields used cover many organs and tissues. CSI is routinely used in the treatment of medulloblastomas, a relatively common tumour type that can occur across all ages of development which also facilitates data availability. Treatment positioning is supine and consistent across patients, reducing variability in pose. In this cohort the gross tumour is usually resected prior to radiotherapy and hence not visible on CT, minimising potential issues that variable gross tumour positions could cause on the atlas construction. A limitation is that shunts and, particularly for younger patients, intubation are commonly used, adding external elements and artefacts to the CTs. While we have demonstrated that other cohorts can be satisfactory overlaid on the template CT, registration quality metrics were higher for CSI subjects likely due to the increased similarity in terms of setup and presentation. The patients in non-CSI cohorts were also younger on average and hence it is possible that larger deformations had to be captured. Other cohorts will also have unique characteristics not commonly found in the CSI cohort. For example, excessive gas in the bowel is observed commonly in abdominal neuroblastoma patients (Lim *et al* 2020), increasing the challenges in co-registering these images to a CSI-based template.

The methodology’s success in spatially normalising radiotherapy data depends on the accuracy achieved in inter-subject DIR. The paediatric population is particularly challenging to co-register. Large deformations must be captured to co-register subjects across development stages, which poses a complex challenge to DIR due to inter-subject variability across sex, age, height, weight, internal anatomy and abnormalities caused by disease. These challenges differ between anatomical regions. Volumes in the abdomen and pelvis are highly deformable and, due to physiological motion, one-to-one mapping does not always exist, making the registrations very challenging. Indeed, we found better spatial mapping for organs in head and thorax (DSC of 0.95 ± 0.05, 0.85 ± 0.04 and 0.96 ± 0.01 for CNS, ocular globes and lungs) than in other regions (0.91 ± 0.03, 0.83 ± 0.06 and 0.65 ± 0.16 for liver, kidneys and bladder). The poorest performance was in the bladder, a highly deformable organ: 50% of the subjects had a DSC below 0.7. For reference, American Association of Physicists in Medicine Radiation Therapy Committee Task Group 132 suggest a DSC of 0.8–0.9 as being good performance for image registration (Brock *et al* 2017). However, it must be noted that DSC is higher for larger volumes and interpretation of the absolute values must always consider the absolute organ volumes. RDA and DOO ranged between 0.02 ± 0.04–0.29 ± 0.28 and 0.36 ± 0.18–0.92 ± 0.07, respectively, for the same contours. These are comparable to values reported in other studies. In a recent study, Pilia *et al* report DSC of 0.80 ± 0.11, 0.44 ± 0.23, and 0.58 ± 0.14 for liver, kidneys and bladder, respectively, when using Elastix groupwise to co-register adults whole body MRIs (Pilia *et al* 2019). Acosta *et al* report RDA and DOO of 0.09 ± 0.05 and 0.64 ± 0.1 for spatial normalisation of rectum dose (Acosta *et al* 2013). Monti *et al* report a DOO in the range 0.39 ± 0.11–0.58 ± 0.10 for dose to brain sub-volumes (Monti *et al* 2020).

We consider our results promising, particularly when taking into consideration that we are using a general-purpose registration methodology, the task of inter-subject registration is very challenging, and the fact that we are dealing with whole-body images. Nevertheless, more specialised approaches should be explored in the future, particularly to improve matching in highly deformable organs (e.g. bladder) or when there is no true one-to-one mapping (e.g. regions of bowel gas). For example, by using additional *a priori* structural information (i.e. landmarks or contours) to guide the registration such that large local deformations can be better captured (Johnson and Christensen 2002, Rivest-Hénault *et al* 2014). Further work is also needed to evaluate the atlas constructed using more comprehensive datasets, with more organs and numbers of patient per cohort, ideally from multiple institutions.

One of the most promising applications of spatial normalisation is to develop voxel-based risk models of late effects that account for heterogeneous spatial radiosensitivity, which can potentially be used to develop personalised risk-guided therapies. This is an emerging area in radiation toxicities research (Palma *et al* 2020). Other groups have investigated voxel-based analysis to identify radiosensitive subregions of organs (such as bladder, rectum, lungs and head and neck) in adult cohorts, which can then be avoided during RT planning (Acosta *et al* 2013, Palma *et al* 2016, Monti *et al* 2017, McWilliam *et al* 2017, Beasley *et al* 2018). Palma *et al* introduced recently the concept of comprehensive normal tissue complication probability (NTCP) models that include full spatial information of the dose distributions (Palma *et al* 2019a). The present study is the first step toward voxel-based analysis in radiation-induced toxicities after paediatric radiotherapy. Our next step is to use the proposed methodology to explore the dose-response relationships for paediatric late effects.

The spatial normalisation process was evaluated in our study at the organ level, which is the level of accuracy historically used in radiation-induced toxicity analysis. Our results indicate similar NTCP models can be generated using the common reference or native spaces. For voxel-based analysis applications, validation of spatial mapping at sub-structures and/or voxel level is required but it is a challenging problem, particularly for homogeneous organs with few imaging features. Further work is therefore required to evaluate and improve registrations at finer resolutions. For example, this could be done by evaluating the accuracy of mapping anatomical landmarks, or by dividing organs into well-defined sub-structures that can be analysed separately. Improving localised mapping is increasingly relevant for clinical endpoints such as brain injury (Gunther *et al* 2015, Viselner *et al* 2019), lung fibrosis (Veiga *et al* 2018) and heart failure (McWilliam *et al* 2017). We recommend that in clinical studies investigating organ-specific end-points additional validation is performed accordingly. Better soft tissue mapping may be achievable by incorporating complementary multimodal imaging such as MRI (Monti *et al* 2020), or by digitally enhancing the CT images to improve contrast. Furthermore, we would like to highlight that achieving adequate voxel-level mapping allows one to potentially develop radiation-induced toxicity predictive models that consider simultaneously with the local dose, the localised tissue radiosensitivity which can be measured with co-registered multimodal functional imaging (Yankeelov *et al* 2014). In the paediatric population, accounting for patient-specific radiosensitivity is particularly important as spurts of growth are occurring as part of the normal development into adulthood.

While the methodology is not specific to this clinical endpoint and can be adapted to other endpoints, in our opinion it is very promising in the study of radiation-induced SMNs. The use of the template CT for analysis addresses some of the challenges associated with this end-point:

(1) Spatial normalisation brings the opportunity to understand the SMN dose-response as function of the local dose instead of dose to volume (e.g. average dose) and potentially identify sub-regions of increased radiosensitivity.
(2) The methodology generates standardised whole-body organ segmentations that are often missing from routine clinical data (i.e. atlas-based segmentation). This has advantages even for traditional DVH-based modelling where manual segmentation becomes prohibitive for large numbers of subjects. Manual segmentation is associated with variability between clinicians and is challenging to deploy practically on larger datasets as several organs relevant to SMN risk are not segmented clinically due limited clinical resources.
(3) The template CT can be used to account for missing anatomical information, as it can be used to estimate out-of-field doses and volumes (i.e. population-representative virtual phantom). Typically, the planning CT images do not cover the whole-body (only treated regions), which complicates studying out-of-field effects. The template CT built from the CSI cohort covers all organs and therefore can be used as radiotherapy-specific synthetic phantom to estimate of anatomy and out-of-field doses. In this case, it is increasingly important to investigate age and gender-specific templates. The use of computational and/or physical phantoms is common in radiation dosimetry, although these are usually built from healthy individuals (Christ *et al* 2009, Segars *et al* 2009, Lee *et al* 2015, Xie *et al* 2017).
(4) The template CT is whole-body and hence allows us to harness routine dose-volume information from patient groups which were irradiated at different sites, making the most of partial information and allowing to understand the dose-response at different dose levels (i.e. inside the RT target, near-target and out-of-field).

Choosing a representative common reference space is a key step for spatial normalisation. The template CT proposed was constructed using groupwise image registration to reduce bias associated with the choice of the common reference space in subsequent analysis. The choice of image used to initialise the atlas construction process can still bias the final template generated (Agier *et al* 2020); however, the differences we found when varying the initialisation were small. To the best of our knowledge, using population-specific average atlases for radiotherapy applications, as in our study, had not been investigated before in the literature of radiation-induced toxicities. The typical approach is to define an individual from the population as the single template. This can be empirically performed—for example, manually by visual inspection (Beasley *et al* 2018) or choosing a subject with mean/median anatomical features (Palma *et al* 2016, Monti *et al* 2017, Mylona *et al* 2019). We report poorer anatomical and dose mapping metrics using this simpler approach than for the average atlas. Another method is to use less biased methods of identifying the population’s most representative individual—for example, using clustering approaches (Acosta *et al* 2013, Marcello *et al* 2020). There are a variety of methods proposed for optimal atlas selection developed in the context atlas-based segmentation (Rohlfing *et al* 2004, Aljabar *et al* 2007, Zhou *et al* 2014, Iglesias and Sabuncu 2015). Others have used well-established anatomical atlases developed for neuroimaging applications (Monti *et al* 2020) or virtual anatomies (Palma *et al* 2019b). These templates are theoretically easier to share between institutions and facilitate standardisation of how spatial analysis is performed across studies; however, they are not necessarily representative of the populations analysed. With this in mind, we aim to make our model available in the future to facilitate other studies in paediatric late effects (https://cmic-rt.github.io/RT-PAL/). The bias in atlas selection and subsequent voxel-based analysis of toxicity can be mitigated by repeating analysis on multiple references to verify if similar spatial patterns arise even when the common reference space varies (Dréan *et al* 2016, Marcello *et al* 2020, McWilliam *et al* 2020).

We used a general-purpose, well-established groupwise registration tool, tailored to better deal with the challenges in co-registering the CSI paediatric cancer population. More efficient approaches could be explored, both in terms of memory requirements and to better deal with co-registering heterogenous datasets. Groupwise image registration is a popular methodology in human brain studies (Dickie *et al* 2017); whole-body studies like ours are still rare due to challenges in registering heterogeneous large datasets of high resolution images (Pilia *et al* 2019, Agier *et al* 2020). While the template constructed effectively represented both genders and a wide range of ages for demonstration purposes, it is admittedly a simplified approach not able to fully account for the anatomical variation in heterogeneous populations. Multiple (age and gender-specific) templates may be constructed with larger datasets. Accounting for anatomical differences between genders is relevant as side-effects can be gender-specific (for example, second breast cancers (Inskip *et al* 2009)). The benefits of age-appropriate atlases have been demonstrated in neuroimaging applications (Fonov *et al* 2011). While the quality of mapping may be improved by splitting the population into sub-groups using several, more refined atlases, this will also reduce how generalisable the methodology and subsequent findings are. Hence a single representative template is an attractive approach, particularly in rare populations (such as paediatric cancers) where it is more challenging to gather large datasets. Alternatively, atlas synthesis has been proposed using hierarchical imaging clustering to form a pyramid of classes (Wang *et al* 2010). Alternative strategies may help with the challenges in co-registering whole-body images and scaling to larger datasets, by using deep-learning to speed up the processes (Ahmad *et al* 2019) or by avoiding dense registration (Agier *et al* 2020).

Despite the associated challenges, methodologies focused on whole-body imaging have the potential of enabling risk prediction in big data studies. Examples of the potential applications that leverage three-dimensional whole-body population data are discussed in detail by Strand *et al* and include anomaly detection, group comparisons, longitudinal analysis and correlation analysis (Strand *et al* 2017). Similar ideas were recently explored in the context of radiotherapy toxicity predictive modelling for liver stereotactic ablative radiotherapy (Ibragimov *et al* 2018, 2019). These studies harnessed the potential of artificial intelligence and spatial normalisation to a common reference space to combine complex three-dimensional imaging and non-imaging data to build predictive models of radiotherapy outcomes.

Finally, detailed long-term data collection is essential to understand and minimise adverse effects of radiotherapy. Single institutions have limited ability to gather adequate data due to the rarity of childhood cancers. Late effects such as SMNs have long latency periods which make data collection challenging (Armstrong *et al* 2009, Bhakta *et al* 2017). The need for comprehensive, multi-institutional collection of dosimetry and follow up data is recognised by the paediatric radiotherapy community, with on-going initiatives to combine efforts to accelerate outcomes-based research toward patient benefit (Berrington de Gonzalez *et al* 2017). An example of such initiatives is the paediatric proton/photon consortium registry, a consented registry with 15 institutions that has been collating detailed baseline, treatment and follow-up information since 2012, including planning CT images and dosimetry (Lawell *et al* 2019). The methodology developed in this work aims to leverage complex 3D data and facilitate analysis in such emerging rich datasets and clinical trials data.

## 5. Conclusions

In this work, we proposed and evaluated atlas construction and spatial normalisation in paediatric radiotherapy CTs. An atlas-based template CT model representative of the paediatric cancer population was developed using groupwise image registration. Spatial normalisation to this template CT was evaluated with promising results, indicating it is possible to spatially standardise the paediatric radiotherapy populations despite considerable variability in age and gender. The proposed framework leverages DIR to facilitate modelling and validation of predictive models of radiation-induced late effects after childhood cancer radiotherapy. This study is the first step toward voxel-based analysis in radiation-induced toxicities following paediatric radiotherapy.
